# One-visit RCT of Maxillary Incisors with Extensive Inflammatory Root Resorption and Periradicular Lesions: A Case Report

**Published:** 2011-05-15

**Authors:** Saeed Asgary, Maryam Ahmadyar

**Affiliations:** 1. Department of Endodontics, Iranian Center for Endodontic Research, Dental Research Center, School of Dentistry, Shahid Beheshti University of Medical Sciences, Tehran, Iran.; 2. Reasearcher, Iranian Center for Endodontic Research, Dental Research Center, School of Dentistry, Shahid Beheshti University of Medical Sciences, Tehran, Iran.

**Keywords:** Endodontic, Lesion, Periradicular, Root Canal Therapy, Root Resorption

## Abstract

Inflammatory external root resorption (IERR) is a pathological phenomenon of microbial origin. This study reports a case of external apical inflammatory root resorption of maxillary incisors associated with periradicular lesions in a 22 year old female, which was successfully treated by one-visit root canal therapy (RCT). Radiographic investigation revealed periapical radiolucencies in the upper incisors associated with varying degrees of external inflammatory root resorption of teeth 12, 21 and 22. One-visit RCT of all involved teeth was carried out. Access cavities were permanently restored after 1 week. Clinical and radiographic examinations at 1 day, 1 week and 14 month follow-up demonstrated complete resolution of patient's signs/symptoms. The incisors were fully functional, and complete bone healing of the apical radiolucencies had taken place. The treatment outcomes demonstrated that IERR associated with periradicular lesions can respond successfully to one-visit RCT when conducted with adequate disinfection and a satisfactory coronal seal. Hence, one-visit RCT may be a good alternative to multiple-visit RCT involving intra-canal medicaments.

## INTRODUCTION

Physiological root resorption is a process that involves resorptive activity followed by periods of attempted repair [1]. The process of inflammatory external root resorption (IERR) in the permanent dentition is usually pathological [2] and is most frequently associated with inflammation of microbial origin [3]. Several different types of resorption are recognized: some are isolated to one tooth and slow spreading; others are rapid, aggressive and may involve several teeth [4].

In IERR, loss of the cementum layer allows communication between the root canal and the periodontal ligament (PDL) through the open dentinal tubules. The areas affected will be in the region of the main apical foramen or lateral canal openings [4]. Bacteria remaining in the dentinal tubules can provide a reservoir for re-infection [5], and therefore, it is critical to reduce/eliminate pulpal bacteria and endotoxin which stimulate the resorptive process [6].

Current recommendation is to fill teeth radiographically diagnosed with IERR with calcium hydroxide (CH) over long-term [7]. However, despite the current data showing a high success rate for long-term CH therapy [8], disadvantages include the time consuming nature of IERR treatment, weakening of the root structure and necrotizing effects of CH [9]. In addition, the degree of antibacterial activity of CH is still controversial [10].

Researchers have attested the inefficacy of CH in eliminating bacteria inside dentinal tubules. In particular, CH has been shown to be ineffective against Enterococcus faecalis [11] which is an important agent in persistent endodontic infections [12].There are certain advantages in one-visit root canal therapy (RCT): it is faster, better tolerated by patients and avoids recontamination of root canals between appointments. However, there is still a school of thought that opposes one-visit endodontic treatment of necrotic pulps [13].

Online search of endodontic literature did not reveal any studies on one-visit RCT of IERR. This case report presents successful one-visit treatment of four anterior teeth, three of which presented with IERR secondary to widespread caries and pulpal inflammation/infection.

## CASE REPORT

**Figure 1:**
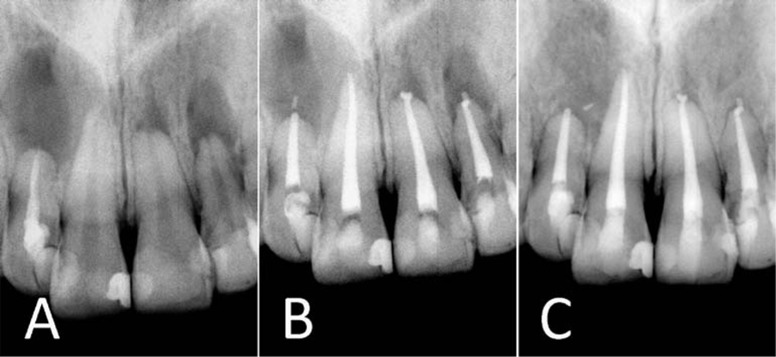
A) Pre-operative radiograph; B) Immediate postoperative radiograph; C) 14-month follow-up radiograph

A 22-year old female was referred to a private clinic with a chief complaint of occasional pain/discomfort in the maxillary incisors. There was no significant medical history. Clinical examination revealed a fully dentate patient with inadequate level of oral hygiene. All four maxillary anterior teeth were heavily restored and were slightly tender to percussion and palpation. Radiographic investigation revealed periapical lesions associated with varying degrees of IERR of teeth 12, 21 and 22, and previous RCT of tooth 12 (Figure 1A). Electric pulp tests were performed and were negative in all of the maxillary incisors. Following thorough explanation of various treatment procedures, full-written consent was obtained from the patient. We decided to complete endodontic treatment of all four maxillary incisors by one endodontist in a single session.

Local anesthesia was administered using 2% Lidocaine and 1:80,000 epinephrine (Darou Pakhsh, Tehran, Iran). Following isolation, access cavities were prepared using a diamond fissure bur (Diatech, Heerbrugg, Switzerland). Working length determined radiographically. Instrumentation of the canals was performed by gates glidden and hand K-files (Mani, Japan) using the step-back technique. Copious irrigation using 5.25% NaOCl solution was carried out. The canals were dried using paper points (Aria Dent, Tehran, Iran) and obturated by lateral condensation technique with gutta-percha (Aria Dent, Tehran, Iran) and Roth endodontic sealer (Roth's 801 sealer, Roth Int., Chicago, IL, USA). Access cavities were temporized by using Cavit (Asia Chemi Teb Co, Tehran, Iran) (Figure 1B). The patient was reviewed after 1 day and one week. In the absence of symptoms at the one week review appointment, the access cavities were restored with adhesive restorations.

The patient was recalled 14 months postoperatively. All four incisor teeth were asymptomatic, and no clinical signs/symptoms of infection or inflammation were observed. The IERR was considered healed if the PDL was re-established and the resorptive process arrested. Radiographic examination at 14 month follow-up revealed successful treatment, i.e. complete healing of the apical lesions accompanied by arrest of the IERR process (Figure 1C).

## DISCUSSION

In this study, an attempt was made to treat IERR by one-visit RCT without inter-appointment medication, which demands recurrent visits and high levels of patient cooperation. This treatment modality is supported by the results of a recent Cochrane systematic review which revealed that one- and multiple-visit RCTs had equivalent success rates [14].

The etiology of IERR is thought to be due to periodontal injury and the concurrent presence of an infected pulp inducing osteoclast resorption [15]. IERR usually occurs without any clinical signs/symptoms and forms part of the periapical pathology associated with apical periodontitis [16]. In order to arrest inflammation and allow regeneration of the PDL, treatment of IERR should be based on reduction of bacterial numbers and their by-products from the root canals and dentinal tubules [17]. A Cochrane systematic review revealed that there is no reliable base of evidence regarding the most suitable protocol of treating IERR, and in most cases treatment is based on clinicians' experiences and patient related factors [18]. At present, the preferred treatment protocol for IERR consists of a short-term dressing of a creamy paste of CH (one month), followed by a long-term dressing of densely packed CH in order to kill the bacteria inside the dentinal tubules and neutralize endotoxins [19]. The main mechanism of action of CH is to provide an alkaline pH via its disassociation into Ca+ and -OH ions which can diffuse through dentinal tubules [4].

Treatment of IERR using CH dressing has a number of disadvantages including necrotizing effects, increasing dentin brittleness and unpredictable antibacterial efficacy. CH use in teeth with damaged cementum is contraindicated due to the necrotizing effects of CH on PDL cells [9]. In addition, the long-term presence of CH in root canals can increase brittleness of the root dentin [20]. Moreover, calcium hydroxide does not kill all bacterial species associated with infected root canals [21]. Several studies have attested the inefficacy of CH in eliminating bacteria inside dentinal tubules, as it has been found to be ineffective in sterilizing dentine, preventing secondary infection [22], and eliminating E. faecalis even after extended periods of treatment [22][23][24].

Studies have demonstrated that correct root canal preparation, accompanied by abundant antibacterial irrigation, is able to drastically reduce the number of microorganisms [25]. The successful outcome of this case indicates that by carrying out timely chemomechanical preparation with copious 5.25% NaOCl irrigation, it is possible to eradicate bacteria and their by-products in order to allow healing of the periradicular lesions. NaOCl has well-known bactericidal properties and as an endodontic irrigant it bleaches, deodorizes, dissolves tissue and disinfects the root canal system [26]. NaOCl is usually used in a concentration range of 0.5-5.25%, though previous studies have confirmed that exposure to just 0.5-1% NaOCl solution was lethal to bacteria [27].

Another important variable in this case report was the working length of the root canals. It is known that in cases with periradicular radiolucencies, preparation and obturation of root canals as close as possible to the apical terminus is desirable [28]. This was carried out in all four incisors, thus allowing removal of microorganisms persisting in the apical portion of the canals and leading to a successful treatment outcome.

## CONCLUSION

The results of this case report demonstrated that inflammatory external root resorption associated with periradicular lesions can respond successfully to one-visit RCT. In general, the successful results of this case indicate that one-visit RCT using adequate chemomechanical preparation with 5.25% NaOCl irrigation and a satisfactory coronal seal presents an alternative treatment modality, although further studies are required to confirm this.
